# Suicide mortality following the implementation of tobacco packaging and pricing policies in Korea: an interrupted time-series analysis

**DOI:** 10.1186/s12916-024-03372-5

**Published:** 2024-04-29

**Authors:** Taiyue Jin, Juhee Seo, Shinhee Ye, Seulbi Lee, Eun Young Park, Jin-Kyoung Oh, Changwoo Han, Byungmi Kim

**Affiliations:** 1 Division of Cancer Prevention, National Cancer Control Institute, National Cancer Center, 323 Ilsan-Ro, Ilsandong-Gu, Goyang, Gyeonggi 10408 South Korea; 2 Vital Statistics Division, Statistics Korea, Daejeon, South Korea; 3 Occupational Safety and Health Research Institute, Korea Occupational Safety and Health Agency, Incheon, South Korea; 4 Department of Big Data Strategy, National Health Insurance Service, Wonju, South Korea; 5 Department of Preventive Medicine, Korea University College of Medicine, Seoul, South Korea; 6 Department of Cancer Control and Population Health, Graduate School of Cancer Science and Policy, National Cancer Center, Goyang, South Korea; 7 Department of Preventive Medicine, Chungnam National University College of Medicine, Daejeon, South Korea

**Keywords:** Tobacco use, Policy, Suicide, Interrupted time-series analysis, Korea

## Abstract

**Background:**

To prevent tobacco use in Korea, the national quitline number was added to tobacco packages in December 2012, tobacco prices were raised by 80% in January 2015, and graphic health warning labels were placed on tobacco packages in December 2016. This study evaluated the association of these tobacco packaging and pricing policies with suicide mortality in Korea.

**Methods:**

Monthly mortality from suicide was obtained from Cause-of-Death Statistics in Korea from December 2007 to December 2019. Interrupted time-series analysis was performed using segmented Poisson regression models. Relative risks (RRs) and 95% confidence intervals (CIs) were calculated adjusted for suicide prevention strategies.

**Results:**

Suicide mortality was 20 per 1,000,000 in December 2007 and showed a downward trend over the study period. After the implementation of tobacco packaging and pricing policies, suicide mortality immediately declined by − 0.09 percent points (95% CI =  − 0.19 to 0.01; *P* > 0.05) for the national quitline number, − 0.22 percent points (95% CI =  − 0.35 to − 0.09; *P* < 0.01) for tobacco prices, and − 0.30 percent points (95% CI =  − 0.49 to − 0.11; *P* < 0.01) for graphic health warning labels. The corresponding RRs for these post-implementation changes compared with the pre-implementation level were 0.91 (95% CI = 0.83 to 1.00), 0.80 (95% CI = 0.70 to 0.91), and 0.74 (95% CI = 0.61 to 0.90), respectively. Significant associations between tobacco control policies and suicide mortality were observed even when stratified by sex and region.

**Conclusions:**

The findings of this study provide new evidence for an association between tobacco control policies and deaths by suicide. An array of effective tobacco control policies should be considered for prevention programs targeting suicide.

## Background

In accordance with the World Health Organization (WHO) global report on tobacco use prevalence trends in 2000–2025, the global prevalence of smoking decreased from 26.9% in 2000 to 17.0% in 2020 among people aged 15 years and older [1]. The WHO report also notes that the slowest decline in smoking prevalence during these two decades—at 4.8%—occurred in the Western Pacific Region. Although the prevalence of smoking in Korea has been slowly waning since the early 2000s, the rate in 2020 was 20.6% [2], which is still higher than the global average of 17.0%, especially among men (34.0% in Korea vs. 28.9% worldwide).

To address the global response to the tobacco epidemic, the World Health Assembly adopted the WHO Framework Convention on Tobacco Control (FCTC) in 2003 [3]. In 2008, the WHO FCTC introduced the MPOWER policy package, which includes six comprehensive demand-reduction measures to protect people from tobacco exposure, support for quitting smoking, and prevent smoking initiation [4]. As of 2020, 146 countries had adopted at least 1 MPOWER strategy [5]. A study using data from 63 countries that adopted the MPOWER strategies suggested that they have had a beneficial impact on global smoking prevalence and intensity [6]. Another study found that implementing the highest level of demand-reduction measures was responsible for 2.6 percent points drop in smoking prevalence across 126 countries [7].

In Korea, text-only health warning labels were first introduced on tobacco packages in 1976. In the following decades, the government implemented a series of actions regarding tobacco packaging and pricing (Table 1). In December 2012, the national quitline number was added to tobacco packages. In January 2015, tobacco prices in Korea increased by 80%, from 2500 Korean won (KRW) to 4500 KRW for a pack of cigarettes. In December 2016, graphic health warning labels, including a series of pictures of the harms of smoking (i.e., lung cancer, larynx cancer, oral cavity cancer, heart disease, stroke, children’s second-hand smoke, prenatal smoke, erectile dysfunction, skin aging, and premature death), were placed on the packaging of tobacco products. Previous studies evaluating the effectiveness of these interventions indicated that awareness of the quitline number doubled after its addition to tobacco packages [8] and that the proportion of quit-attempters among current smokers increased after tobacco prices increased and graphic health warning labels were placed on tobacco packages [9, 10].

**Table 1:** Key policies on tobacco packaging and pricing in Korea

Date of implementation	Key policy
July 1976	Text-based health warning labels added to tobacco packages
September 1995	National Health Promotion Act enacted
January 2003	Tar and nicotine contents listed on tobacco packages
December 2004	Tobacco prices increased by 25% (from 2000 to 2500 KRW)
December 2008	The six carcinogenic substances in tobacco (naphthylamine, nickel, benzene, vinyl chloride, arsenic, and cadmium) listed on tobacco packages
December 2012	National quitline number added to tobacco packages
January 2015	Tobacco prices increased by 80% (from 2500 to 4500 KRW)
December 2016	Graphic health warning labels placed on tobacco packages
December 2018	Graphic health warning labels placed on e-cigarette packages

*KRW* Korean won

In addition to smoking, the persistent increase in deaths by suicide, suicide attempts, and suicidal ideation is also a global public health concern. A WHO report stated that an estimated 703,000 people died of suicide in 2019, which is equivalent to 1 death by suicide every 40 s [11]. In the same year, the suicide rate in Korea (24.6 per 100,000) was more than twice the average of Organisation for Economic Cooperation and Development (OECD) countries (11.0 per 100,000), ranking the highest since the early 2000s [12].

Previous observational studies showed an inverse association between quitting smoking and risk of suicide [13, 14]. In a previous ITS analysis, suicide mortality reduced following the increase of tobacco taxes and strengthen of smoke-free air laws in the United States (US) [15]. In addition, in that study, the inverse association between tobacco control policies and suicide was more obvious among individuals with the highest quartile of smoking prevalence (29.7%) than those with the lowest quartile (10.8%). However, to the best of our knowledge, no study has evaluated the impact on suicide of key demand-reduction measures regarding tobacco packaging and pricing, which may decrease smoking prevalence by encouraging quitting and preventing smoking initiation [16]. Hence, in this study, we performed ITS analysis to examine whether the implementation of tobacco packaging and pricing policies affected suicide mortality at a population scale in Korea.

## Methods

### Study design

An ecological study with an ITS design was conducted in this study. A time series data was used to detect changes in suicide mortality trends after the implementation of tobacco packaging and pricing policies in Korea. Comparisons were based on a counterfactual scenario, hypothesizing that the trend remained unchanged until implementation occurred [17]. To ensure sufficient detection power, the pre-implementation period was defined as 5 years before the first tobacco control policy (i.e., December 2012) [18]. Due to the global COVID-19 pandemic, the post-implementation period ended 3 years after the last tobacco control policy (i.e., December 2016). As a result, the study period was December 2007 to December 2019.

### Data collection

Monthly suicide mortality data from December 2007 to December 2019 was acquired from the Cause-of-Death Statistics tracked by Statistics Korea, the government organization that manages national statistics in Korea [19]. The underlying individual-level causes of death are collected from death certificates filed in local administration offices. Cause-of-Death Statistics provide nationwide information on deaths, including the number of deaths, causes of death, geographical distribution of deaths, and ranking of causes of death since 1982. The annual report on the Cause-of-Death Statistics is published in September of the following year.

In the Cause-of-Death Statistics, the underlying causes of death were classified based on the International Statistical Classification of Diseases and Related Health Problems, version 10 (ICD-10) [20], and the Korean Standard Classification of Diseases and Causes of Death, version 7 (KCD-7), which was adapted to fit the disease status and medical conditions in Korea [21]. In this study, suicide was defined as death by intentional self-harm (X60–X84).

On the other hand, age-standardized annual smoking prevalence from 2007 to 2019 was derived from the Korea National Health and Nutrition Examination Survey (KNHANES), a national representative health survey conducted annually in Korea [22, 23].

### Statistical analysis

**Fig. 1:**
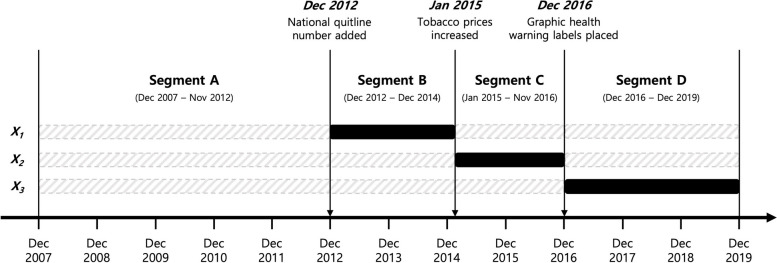
Variables indicating pre- and post-implementation periods of the tobacco control policies. The *X*_1_, *X*_2_, and *X*_3_ were the dummy variables included in the segmented Poisson regression model. The dummy variables were coded as 1 for the periods after each tobacco control policy implemented (black filled boxes) and 0 for the remaining periods (gray dashed boxes)

Segmented Poisson regression models with an ITS design were applied to analyze deaths count data. The implementation dates of the three tobacco packaging and pricing policies—national quitline number added to tobacco packages in December 2012, increase in tobacco prices in January 2015, and graphic health warning labels placed on tobacco packages in December 2016—were modeled to evaluate the association with monthly mortality from suicide compared with the trend under the counterfactual scenario [24]. Data were divided into four segments: segment A from December 2007 to November 2012, segment B from December 2012 to December 2014, segment C from January 2015 to November 2016, and segment D from December 2016 to December 2019. The unadjusted ITS analysis in this study (model 1) was specified as:

$$\text{log}\left(Y\right)=\beta_0+\beta_1T+\beta_2X_1+\beta_3X_2+\beta_4X_3+\beta_5\left(T-T_0\right)\cdot X_1+\beta_6\left(T-T_0\right)\cdot X_2+\beta_7\left(T-T_0\right)\cdot X_3+\varepsilon$$

where *Y* is the monthly mortality from suicide (an offset variable of total population was used to fit the Poisson distribution); *T* is the time elapsed from December 2007 to December 2019; *T*_0_ is the time when the policy implementation began; *X*_1_, *X*_2_, and *X*_3_ are dummy variables indicating the pre- and post-implementation periods of the three tobacco control policies (Fig. 1); *β*_0_ is the baseline level of suicide mortality before implementation of the first policy; *β*_1_ is the change in suicide mortality with the increment in time unit; *β*_2_, *β*_3_, and *β*_4_ are level changes in suicide mortality from pre- to post-implementation of the three policies; *β*_5_, *β*_6_, and *β*_7_ are the trend (slope) changes in suicide mortality from pre- to post-implementation of the three policies; and *ε* is an error term.

We calculated monthly mortality from suicide (per 1,000,000) by dividing the number of deaths per month by the size of the population at the midpoint of the year. As the ITS design is mainly affected by time-varying confounding factors such as population age distribution, age standardization was performed based on the 2005 Korean population (*n* = 36,820,786.5) using the direct standardization method [25]. To rule out the over-dispersion issue of time series data, which do not fit the assumption that the variance is equal to the expected count in a Poisson distribution, a quasi-Poisson model was employed (model 2) [24, 26]. We also controlled for seasonality, a time-varying confounding factor, using a Fourier term (model 3) [26]. Furthermore, we adjusted for suicide prevention strategies implemented during the study period (model 4): installing lifeline phone booths in places where suicides occur frequently in July 2011, developing suicide education campaigns in January 2012, and providing medical care costs for suicide attempters admitted to an emergency department in January 2016 [27].

Subgroup analyses according to sex and region were performed. Korean cities with a population of more than 1 million (i.e., Seoul, Busan, Daegu, Incheon, Gwangju, Daejeon, and Ulsan) were categorized as urban areas, and other regions were classified as rural areas. Relative risks (RRs) and 95% confidence intervals (CIs) were calculated by exponentiating the Poisson regression coefficients. To visualize the trend changes in suicide mortality after the implementation of tobacco packaging and pricing policies, plots were generated based on model 3. All analyses were conducted using SAS version 9.4 (SAS Institute Inc., Cary, NC, USA) and R statistical software version 4.2.0 (R Foundation for Statistical Computing, Vienna, Austria).

## Results

### Smoking prevalence and suicide mortality in Korea

**Fig. 2:**
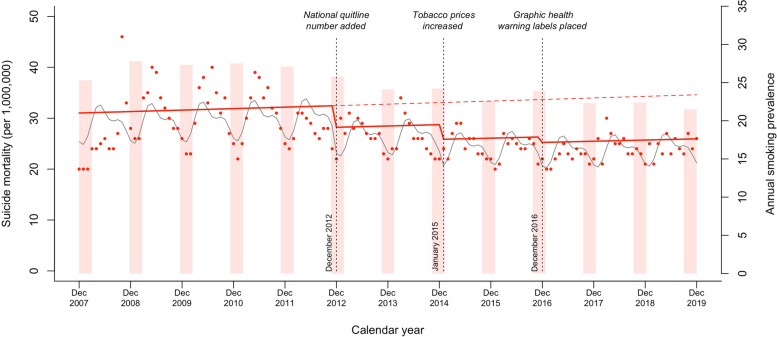
Changes in suicide mortality after implementation of tobacco packaging and pricing policies in Korea from December 2007 to December 2019. Red dots, monthly suicide mortality rate; red filled boxes, annual smoking prevalence; red solid line, projected trend; red dashed line, counterfactual trend; gray solid line, de-seasonalized trend

From the year 2007 onward, the annual smoking prevalence was highest in 2008 (27.8%) and has gradually declined since then (Fig. 2). It should be noted that the annual smoking prevalence reduced slightly 1 year after each tobacco control policy implemented (i.e., 2013, 2015, and 2017). On the other hand, the trend in age-standardized suicide mortality from December 2007 to December 2019 is also shown in Fig. 2. The baseline suicide mortality rate in December 2007 was 20 per 1,000,000, which was the lowest over the study period. The highest suicide mortality rate, 46 per 1,000,000, occurred in October 2008. Compared with the pre-implementation counterfactual trend, suicide mortality was maintained at a relatively low level after the implementation of tobacco control policies. Nevertheless, the suicide mortality rate was 26 per 1,000,000 in December 2019, which was slightly higher than the baseline. Notably, suicide mortality appeared to have a seasonal pattern, with peaks occurring in spring and summer.

### Changes in suicide mortality

Figure 2 and Table 2 show changes in suicide mortality after the implementation of tobacco packaging and pricing policies. In model 3, which controlled for the methodological issues of over-dispersion and seasonality of time series data, overall suicide mortality, compared with the baseline level, decreased by − 0.14 percent points (95% CI =  − 0.23 to − 0.05; *P* < 0.01), − 0.25 percent points (95% CI =  − 0.37 to − 0.12; *P* < 0.001), and − 0.29 percent points (95% CI =  − 0.45 to − 0.12; *P* < 0.01) after addition of the national quitline number to tobacco packages, increased tobacco prices, and placement of graphic health warning labels on tobacco packages, respectively. RRs compared with the baseline level of suicide mortality were 0.87 (95% CI = 0.79 to 0.95) for the national quitline number, 0.78 (95% CI = 0.69 to 0.88) for tobacco prices, and 0.75 (95% CI = 0.64 to 0.88) for graphic health warning labels. However, in model 4, which adjusted for suicide prevention strategies, only marginally significant changes in suicide mortality were observed after the addition of the national quitline number on tobacco packages (RR = 0.91; 95% CI = 0.83 to 1.00). The trend changes in suicide mortality were not statistically significant for any of the tobacco control policies (data not shown).

**Table 2:** Interrupted time-series analysis of suicide mortality by sex from December 2007 to December 2019 in Korea

		December 2007 to November 2012 (segment A)	December 2012 to December 2014 (segment B)		January 2015 to November 2016 (segment C)		December 2016 to December 2019 (segment D)	
		Baseline level^a^	Level change (segment B vs. A)^a^	Relative risk (segment B vs. A)^b^	Level change (segment C vs. A)^a^	Relative risk (segment C vs. A)^b^	Level change (segment D vs. A)^a^	Relative risk (segment D vs. A)^b^
Overall	Model 1^c^	3.35 (3.26, 3.44)	− 0.16 (− 0.30, − 0.03)*	0.85 (0.74, 0.97)	− 0.27 (− 0.45, − 0.08)**	0.77 (0.64, 0.92)	− 0.33 (− 0.57, − 0.09)**	0.72 (0.57, 0.92)
	Model 2^d^	3.35 (3.28, 3.42)	− 0.16 (− 0.27, − 0.06)**	0.85 (0.77, 0.95)	− 0.27 (− 0.41, − 0.12)***	0.77 (0.66, 0.88)	− 0.33 (− 0.52, − 0.14)**	0.72 (0.60, 0.87)
	Model 3^e^	3.36 (3.30, 3.42)	− 0.14 (− 0.23, − 0.05)**	0.87 (0.79, 0.95)	− 0.25 (− 0.37, − 0.12)***	0.78 (0.69, 0.88)	− 0.29 (− 0.45, − 0.12)**	0.75 (0.64, 0.88)
	Model 4^f^	3.32 (3.25, 3.38)	− 0.09 (− 0.19, 0.01)	0.91 (0.83, 1.00)	− 0.22 (− 0.35, − 0.09)**	0.80 (0.70, 0.91)	− 0.30 (− 0.49, − 0.11)**	0.74 (0.61, 0.90)
Men	Model 1^c^	3.61 (3.53, 3.68)	− 0.14 (− 0.26, − 0.03)*	0.87 (0.77, 0.97)	− 0.27 (− 0.42, − 0.11)**	0.77 (0.66, 0.90)	− 0.34 (− 0.55, − 0.13)**	0.71 (0.58, 0.88)
	Model 2^d^	3.61 (3.55, 3.67)	− 0.14 (− 0.24, − 0.05)**	0.87 (0.79, 0.95)	− 0.27 (− 0.40, − 0.13)***	0.77 (0.67, 0.88)	− 0.34 (− 0.51, − 0.16)***	0.71 (0.60, 0.85)
	Model 3^e^	3.61 (3.56, 3.66)	− 0.13 (− 0.21, − 0.05)**	0.88 (0.81, 0.95)	− 0.26 (− 0.37, − 0.15)***	0.77 (0.69, 0.86)	− 0.32 (− 0.46, − 0.17)***	0.73 (0.63, 0.85)
	Model 4^f^	3.57 (3.52, 3.63)	− 0.08 (− 0.16, 0.01)	0.92 (0.85, 1.00)	− 0.22 (− 0.33, − 0.10)***	0.81 (0.72, 0.90)	− 0.29 (− 0.46, − 0.12)**	0.75 (0.63, 0.88)
Women	Model 1^c^	3.01 (2.91, 3.12)	− 0.20 (− 0.37, − 0.04)*	0.82 (0.69, 0.97)	− 0.27 (− 0.50, − 0.04)*	0.76 (0.61, 0.96)	− 0.31 (− 0.61, − 0.01)*	0.74 (0.55, 0.99)
	Model 2^d^	3.01 (2.93, 3.09)	− 0.20 (− 0.33, − 0.07)**	0.82 (0.72, 0.93)	− 0.27 (− 0.45, − 0.09)**	0.76 (0.64, 0.91)	− 0.31 (− 0.54, − 0.07)*	0.74 (0.58, 0.93)
	Model 3^e^	3.03 (2.96, 3.10)	− 0.17 (− 0.29, − 0.05)**	0.84 (0.75, 0.95)	− 0.23 (− 0.39, − 0.07)**	0.79 (0.67, 0.93)	− 0.24 (− 0.45, − 0.03)*	0.79 (0.64, 0.97)
	Model 4^f^	2.98 (2.90, 3.05)	− 0.13 (− 0.26, − 0.01)*	0.88 (0.77, 1.00)	− 0.25 (− 0.42, − 0.08)**	0.78 (0.66, 0.93)	− 0.33 (− 0.58, − 0.08)*	0.72 (0.56, 0.93)

*RR* relative risk, *CI* confidence interval

^*^P < 0.05

^**^P < 0.01

^***^P < 0.001

^a^Data presented as beta-coefficients (95% CIs)

^b^Data presented as relative risks (95% CIs)

^c^Model 1: univariate Poisson regression model

^d^Model 2: quasi-Poisson regression model adjusted for over-dispersion

^e^Model 3: quasi-Poisson regression model adjusted for over-dispersion and seasonality

^f^Model 4: quasi-Poisson regression model adjusted for over-dispersion, seasonality, and suicide prevention strategies in Korea

### Subgroup analysis by sex

**Fig. 3:**
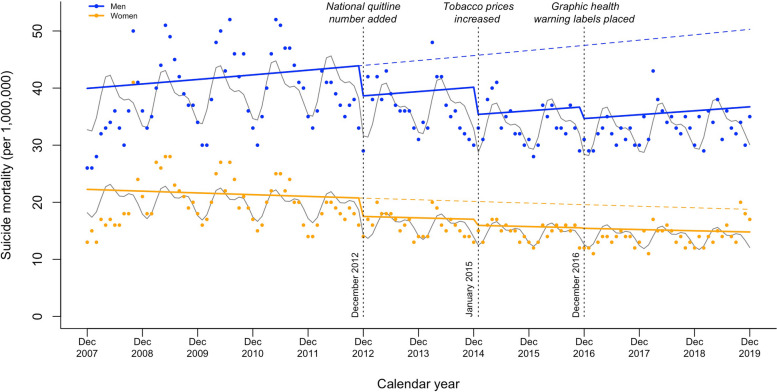
Changes in suicide mortality by sex after implementation of tobacco packaging and pricing policies in Korea from December 2007 to December 2019. Blue and yellow dots, monthly suicide mortality rate in men and women, respectively; blue and yellow solid lines, projected trend in men and women, respectively; blue and yellow dashed lines, counterfactual trend in men and women, respectively; gray solid lines, de-seasonalized trend

When stratified by sex, suicide mortality was higher in men than in women across the entire study period (Fig. 3). After the implementation of tobacco control policies, the trend changes in suicide mortality for men and women were similar to that of the overall sample. Notably, the association between tobacco control policies and suicide mortality was more pronounced in women than in men, with suicide mortality reductions of − 0.08 percent points (95% CI =  − 0.16 to 0.01; *P* > 0.05), − 0.22 percent points (95% CI =  − 0.33 to − 0.10; *P* < 0.001), and − 0.29 percent points (95% CI =  − 0.46 to − 0.12; *P* < 0.01) in men and − 0.13 percent points (95% CI =  − 0.26 to − 0.01; *P* < 0.05), − 0.25 percent points (95% CI =  − 0.42 to − 0.08; *P* < 0.01), and − 0.33 percent points (95% CI =  − 0.58 to − 0.08; *P* < 0.05) in women for national quitline number, tobacco prices, and graphic health warning labels, respectively (Table 2).

### Subgroup analysis by region

**Fig. 4:**
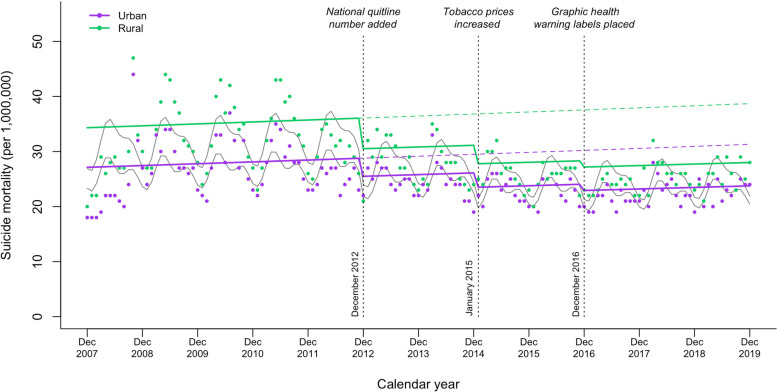
Changes in suicide mortality by region after implementation of tobacco packaging and pricing policies in Korea from December 2007 to December 2019. Purple and green dots, monthly suicide mortality rate in urban and rural areas, respectively; purple and green solid lines, projected trend in urban and rural areas, respectively; purple and green dashed lines, counterfactual trend in urban and rural areas, respectively; gray solid lines, de-seasonalized trend

A higher rate of suicide mortality was observed in rural areas than in urban areas (Fig. 4). Table 3 shows the regional differences in the association between tobacco control policies and mortality from suicide. In urban areas, although there was no significant difference in suicide mortality after the addition of the national quitline number to tobacco packages (RR = 0.93; 95% CI = 0.84 to 1.03), reductions in suicide mortality were observed when tobacco prices increased (RR = 0.80; 95% CI = 0.70 to 0.92) and graphic health warning labels were placed on tobacco packages (RR = 0.72; 95% CI = 0.59 to 0.88). In rural areas, implementation of the three tobacco control policies led to drops in suicide mortality by − 0.12 percent points (95% CI =  − 0.21 to − 0.02; *P* < 0.05) for the national quitline number, − 0.25 percent points (95% CI =  − 0.38 to − 0.12; *P* < 0.001) for tobacco prices, and − 0.32 percent points (95% CI =  − 0.51 to − 0.13; *P* < 0.01) for graphic health warning labels.

**Table 3:** Interrupted time-series analysis of suicide mortality by region from December 2007 to December 2019 in Korea

		December 2007 to November 2012 (segment A)	December 2012 to December 2014 (segment B)		January 2015 to November 2016 (segment C)		December 2016 to December 2019 (segment D)	
		Baseline level^a^	Level change (segment B vs. A)^a^	Relative risk (segment B vs. A)^b^	Level change (segment C vs. A)^a^	Relative risk (segment C vs. A)^b^	Level change (segment D vs. A)^a^	Relative risk (segment D vs. A)^b^
Urban	Model 1^c^	3.24 (3.15, 3.34)	− 0.14 (− 0.28, 0.01)	0.87 (0.76, 1.00)	− 0.24 (− 0.44, − 0.05)*	0.78 (0.65, 0.95)	− 0.31 (− 0.56, − 0.06)*	0.73 (0.57, 0.94)
	Model 2^d^	3.24 (3.18, 3.31)	− 0.14 (− 0.24, − 0.03)**	0.87 (0.78, 0.97)	− 0.24 (− 0.39, − 0.10)**	0.78 (0.68, 0.90)	− 0.31 (− 0.50, − 0.12)**	0.73 (0.61, 0.89)
	Model 3^e^	3.25 (3.19, 3.31)	− 0.12 (− 0.22, − 0.03)*	0.89 (0.80, 0.97)	− 0.23 (− 0.36, − 0.10)**	0.80 (0.70, 0.91)	− 0.28 (− 0.45, − 0.10)**	0.76 (0.64, 0.90)
	Model 4^f^	3.21 (3.14, 3.27)	− 0.08 (− 0.18, 0.03)	0.93 (0.84, 1.03)	− 0.22 (− 0.36, − 0.09)**	0.80 (0.70, 0.92)	− 0.33 (− 0.53, − 0.13)**	0.72 (0.59, 0.88)
Rural	Model 1^c^	3.43 (3.35, 3.52)	− 0.19 (− 0.32, − 0.06)**	0.83 (0.73, 0.94)	− 0.30 (− 0.48, − 0.13)**	0.74 (0.62, 0.88)	− 0.37 (− 0.60, − 0.13)**	0.69 (0.55, 0.87)
	Model 2^d^	3.43 (3.36, 3.50)	− 0.19 (− 0.30, − 0.08)**	0.83 (0.74, 0.92)	− 0.30 (− 0.45, − 0.15)***	0.74 (0.64, 0.86)	− 0.37 (− 0.56, − 0.17)***	0.69 (0.57, 0.84)
	Model 3^e^	3.44 (3.38, 3.50)	− 0.17 (− 0.26, − 0.08)***	0.85 (0.77, 0.92)	− 0.28 (− 0.41, − 0.16)***	0.75 (0.67, 0.85)	− 0.32 (− 0.49, − 0.16)***	0.72 (0.61, 0.85)
	Model 4^f^	3.40 (3.34, 3.46)	− 0.12 (− 0.21, − 0.02)*	0.89 (0.81, 0.98)	− 0.25 (− 0.38, − 0.12)***	0.78 (0.69, 0.89)	− 0.32 (− 0.51, − 0.13)**	0.73 (0.60, 0.88)

*RR* relative risk, *CI* confidence interval

^*^P < 0.05

^**^P < 0.01

^***^P < 0.001

^a^Data presented as beta-coefficients (95% CIs)

^b^Data presented as relative risks (95% CIs)

^c^Model 1: univariate Poisson regression model

^d^Model 2: quasi-Poisson regression model adjusted for over-dispersion

^e^Model 3: quasi-Poisson regression model adjusted for over-dispersion and seasonality

^f^Model 4: quasi-Poisson regression model adjusted for over-dispersion, seasonality, and suicide prevention strategies in Korea

## Discussion

This study used 12 years of nationally representative data to evaluate the effect of tobacco packaging and pricing policies—adding the national quitline number to tobacco packages, increasing tobacco prices, and placing graphic health warning labels on tobacco packages—on suicide mortality in Korea. Even after adjusting for factors that may affect deaths by suicide, suicide mortality declined following the implementation of the three tobacco control policies. This significant association between tobacco control policies and suicide mortality persisted even when stratified by sex and region. The findings of this study can provide new insights into the development of suicide prevention strategies; however, a causal relationship between tobacco control policies and suicide mortality was failed to claim.

The WHO notes the importance of quitline services at the national level (WHO FCTC Article 14) and provides information on toll-free quitline numbers for most countries and territories on their webpage [28]. Evidence from Australia, Canada, Mexico, and New Zealand suggested that adding a quitline number to tobacco packages increases quitline call volumes [29–31], awareness of tobacco risk [32], intention to quit [33], and treatment reach [34, 35]. In Korea, after the addition of the national quitline number on tobacco packages in December 2012, quitline call volumes increased nearly fourfold in 2013 [36]. Raising tobacco prices and taxes also effectively reduces tobacco use [37, 38]. Previous Korean cross-sectional studies demonstrated that the 2015 policy that increased tobacco prices was associated with smoking reduction, quit attempts, and smoking cessation [9, 39]. Likewise, an ITS analysis in Australia showed that smoking prevalence dropped significantly after an immediate 25% tax increase and a pre-announced annual tax increase of 12.5% [40]. In line with findings from real-world data, several simulation studies also reported a negative association between tobacco taxation and smoking prevalence [41, 42]. Previous studies also demonstrated that tobacco package health warnings with graphic images are a more effective tobacco control measure than text-only warnings. A study of 27 European Union countries reported that smokers from countries with graphic health warning labels on tobacco packaging are more likely to attempt to quit than smokers from countries with text-only messages [43]. Similarly, a meta-analysis of 37 studies demonstrated that graphic health warnings elicit more powerful negative emotions and behaviors toward smoking (i.e. attention attracting and holding, aversiveness, negative smoking attitudes, and intention to quit or not start) compared with text-only warnings [44]. Moreover, graphic health warning labels were associated with an up to 19.6% reduction in smoking prevalence in Canada [45].

Approximately 17% of the excess mortality from tobacco use may be explained by causes not considered as common causes of tobacco use [46]. Of these, deaths by suicide account for a significant proportion, with RRs of 3.2–4.4 for current versus never smokers. A large US cohort study found a significantly increased risk of completed suicide among current smokers [47]. Additionally, a meta-analysis of 63 studies indicated that current smokers are at greater risk of suicidal ideation, plans, attempts, and deaths compared with never smokers [48]. Conversely, individuals who quit smoking have a reduced risk of suicide [13, 14]. Furthermore, a study evaluating the association between the duration of abstinence from smoking and suicidal behavior concluded that attempted suicide decreases significantly during a short-term smoking abstinence period of less than 1 year [49].

Previous research illuminated the biological and psychological mechanisms underlying the association between tobacco use and suicide. First, the nicotine in cigarettes decreases levels of serotonin, a neurotransmitter that regulates numerous affective states and behavioral manifestations [50]. Reduced serotonin levels are linked to negative emotions (e.g., mood lability, anxiety, irritability, and depression) and violent behaviors (e.g., impulsivity, hostility, and aggression) that are related to suicidal events. However, evidence from observational studies yields inconsistent results. Most smokers, especially those with pre-existing psychiatric disorders, perceive that smoking benefits their mental health [51–53]. The relief of mental distress, improvement of depressive mood, and alleviation of anxiety after smoking turn them into heavy smokers and make it difficult for them to quit smoking [53–55]. Notwithstanding these findings, it has to be said that mental disorders in regular smokers may be caused by smoking reversely [56, 57]. In a longitudinal cohort study, compared with never smokers, current smokers were found to have significantly higher risk of affective and anxiety disorders [56]. Correspondingly, relief of depression symptoms was also observed when patients with psychiatric problems successfully quit smoking [58]. Second, tobacco use can cause atopic syndrome, which includes asthma, atopic dermatitis, and allergic rhinitis [59–61]. Prolonged pain and disability from these exaggerated immune response–induced diseases could contribute to suicide [60]. Third, tobacco use causes inflammation and oxidative stress [62], which tend to be exhibited at high levels among individuals with suicidal behaviors [63, 64].

According to the Health at a Glance 2021 report by the OECD, the prevalence of tobacco use in Korea has considerably declined during the last decade (from 25.6% in 2009 to 16.4% in 2019) [12]. However, a large gap between men and women still exists. The prevalence of smoking in 2019 was 28.5% for men and 4.4% for women, ranking 9th and 38th, respectively, among the 43 OECD member and candidate countries. In late 2020, the Korea Health Promotion Institute of the Ministry of Health and Welfare announced the National Health Plan 2030, which aims to reduce the prevalence of tobacco use and mortality from suicide by 2030 [65]. To achieve these goals, the Korean government must formulate more comprehensive tobacco control policies, such as further raising tobacco prices or increasing the size of graphic health warning labels on tobacco packages.

We were the first to evaluate the association between tobacco packaging and pricing policies and suicide mortality at the population level in Korea. The findings of this study provide new insights into the management of deaths by suicide via effective tobacco control policies. Our use of the representative data in Korea—Cause-of-Death Statistics—enhances the external validity of our results in adults. Intuitive graphical presentation of the results also improves its interpretability. However, we must also acknowledge several limitations of the study. First, the study design does not allow causal relationships to be drawn between tobacco control policies and suicide mortality. However, because ITS design is not affected by confounding factors with long-term trends, the results of this study provide exploratory evidence for further observational investigations. Second, due to the lack of information on smoking behaviors in the Cause-of-Death Statistics, we cannot determine whether smoking prevalence changed following the implementation of tobacco control policies. Also, the KNHANES only provides annual smoking prevalence data, which limits the ability to identify changes in smoking behaviors. Third, as this study was restricted to adults aged 19 years and older, the results should only be cautiously generalized to child and adolescent smoking and suicide. Finally, because the tobacco control policies of concern in this study were implemented at the national level, we cannot have a comparison with a group that was not subject to the policies. Thus, further investigations on making parallel comparisons at the local level are warranted.

## Conclusions

In conclusion, after the implementation of tobacco control policies in Korea—adding the national quitline number to tobacco packages, increasing tobacco prices, and placing graphic health warning labels on tobacco packages—mortality from suicide declined immediately. To achieve primary prevention of deaths by suicide, more stringent tobacco packaging and pricing policies (e.g., higher tobacco prices, larger graphic health warning labels on tobacco packages, and shorter rotation periods of fear-arousing pictures) should be considered.

## Data Availability

The Cause-of-Death Statistics supporting the conclusions of this article is available online: https://kosis.kr/eng/.

## Abbreviations

CI: Confidence interval
FCTC: Framework Convention on Tobacco Control
ITS: Interrupted time-series
KNHANES: Korea National Health and Nutrition Examination Survey
KRW: Korean won
OECD: Organisation for Economic Cooperation and Development
RR: Relative risk
WHO: World Health Organization

## References

[1] World Health Organization. WHO global report on trends in prevalence of tobacco use 2000–2025, fourth edition. Geneva: World Health Organization; 2021.

[2] Korea Centers for Disease Control and Prevention. Korea Health Statistics 2020: Korea National Health and Nutrition Examination Survey (KNHANES VIII-2). Chengju: Korea Centers for Disease Control and Prevention; 2021.

[3] World Health Organization. WHO Framework Convention on Tobacco Control. Geneva: World Health Organization; 2003.

[4] World Health Organization. MPOWER: a policy package to reverse the tobacco epidemic. Geneva: World Health Organization; 2008.

[5] World Health Organization. WHO report on the global tobacco epidemic 2021: addressing new and emerging products. Geneva: World Health Organization; 2021.

[6] Ngo A, Cheng K-W, Chaloupka FJ, Shang C (2017). The effect of MPOWER scores on cigarette smoking prevalence and consumption. Prev Med.

[7] Gravely S, Giovino GA, Craig L, Commar A, D’Espaignet ET, Schotte K (2017). Implementation of key demand-reduction measures of the WHO Framework Convention on Tobacco Control and change in smoking prevalence in 126 countries: an association study. Lancet Public Health.

[8] Yun EH, Lim MK, Oh JK, Ki IH, Shin SH, Jeong BY (2016). Quitline activity in the Republic of Korea. Asian Pac J Cancer Prev.

[9] Lee B, Seo D-C (2021). Effects of an 80% cigarette price increase on quit attempts, successful quitting and smoking intensity among Korean adult smokers: results from nationally representative longitudinal panel data. Tob Control.

[10] Kim I, Khang Y-H (2020). Differential changes in quitting smoking by daily cigarette consumption and intention to quit after the introduction of a tobacco tax increase and pictorial cigarette pack warnings in Korea, 2013–2017. Drug Alcohol Depend.

[11] World Health Organization (2019). Suicide: one person dies every 40 seconds.

[12] Organisation for Economic Cooperation and Development (OECD). Health at a Glance 2021: OECD Indicators. Paris: Organisation for Economic Cooperation and Development; 2021.

[13] Balbuena L, Tempier R (2014). Independent Association of Chronic Smoking and Abstinence With Suicide. Psychiatr Serv.

[14] Leistikow BN, Shipley MJ (1999). Might stopping smoking reduce injury death risks? A meta-analysis of randomized, controlled trials. Prev Med.

[15] Grucza RA, Plunk AD, Krauss MJ, Cavazos-Rehg PA, Deak J, Gebhardt K (2014). Probing the Smoking-Suicide Association: do smoking policy interventions affect suicide risk?. Nicotine Tob Res.

[16] Janet C-H, Lorraine C, Shannon G, Natalie S, Geoffrey TF (2019). Impact of the WHO FCTC over the first decade: a global evidence review prepared for the Impact Assessment Expert Group. Tob Control.

[17] Shadish WR, C TD, Campbell DT (2002). Experimental and quasi-experimental designs for generalized causal inference. Soc Serv Rev..

[18] Zhang F, Wagner AK, Ross-Degnan D (2011). Simulation-based power calculation for designing interrupted time series analyses of health policy interventions. J Clin Epidemiol.

[19] Statistics Korea. Causes of Death Statistics Daejeon: MicroData Integrated Service. Available from: https://mdis.kostat.go.kr/eng/index.do;jsessionid=jWL1vgFLJZTDbvvFC7QF8WvLkpn6aFsuYHVQ0AqXqdi76sMxbOJKIF4aPFBaJBY9.mdexwas2_servlet_engine2.

[20] World Health Organization. International statistical classification of diseases and related health problems, 10th revision, Fifth edition, 2016. Geneva: World Health Organization; 2015.

[21] Shin H-Y, Kim J, Lee S, Park MS, Park S, Vital Statistics Division Statistics Korea (2020). Cause-of-death statistics in 2018 in the Republic of Korea. J Korean Med Assoc..

[22] Korea Centers for Disease Control and Prevention. Korea National Health and Nutrition Examination Survey (KNHANES) Chengju: Korea National Health and Nutritional Examination Survey. Available from: https://knhanes.kdca.go.kr/knhanes/eng/index.do.

[23] Kweon S, Kim Y, Jang M-j, Kim Y, Kim K, Choi S (2014). Data resource profile: the Korea National Health and Nutrition Examination Survey (KNHANES). Int J Epidemiol..

[24] Bernal JL, Cummins S, Gasparrini A (2017). Interrupted time series regression for the evaluation of public health interventions: a tutorial. Int J Epidemiol.

[25] Lopez Bernal J, Soumerai S, Gasparrini A (2018). A methodological framework for model selection in interrupted time series studies. J Clin Epidemiol.

[26] Bhaskaran K, Gasparrini A, Hajat S, Smeeth L, Armstrong B (2013). Time series regression studies in environmental epidemiology. Int J Epidemiol.

[27] Park SC, Na KS, Kwon SJ, Kim M, Kim HJ, Baik M (2020). “Suicide CARE” (Standardized Suicide Prevention Program for Gatekeeper Intervention in Korea): an update. Psychiatry Investig.

[28] World Health Organization. Toll-free quitlines. Available from: https://www.who.int/campaigns/world-no-tobacco-day/2021/quitting-toolkit/toll-free-quitlines.

[29] Miller CL, Hill DJ, Quester PG, Hiller JE (2009). Impact on the Australian Quitline of new graphic cigarette pack warnings including the Quitline number. Tob Control.

[30] Wilson N, Li J, Hoek J, Edwards R, Peace J (2010). Long-term benefit of increasing the prominence of a quitline number on cigarette packaging: 3 years of Quitline call data. N Z Med J.

[31] Thrasher JF, Osman A, Moodie C, Hammond D, Bansal-Travers M, Cummings KM (2015). Promoting cessation resources through cigarette package warning labels: a longitudinal survey with adult smokers in Canada, Australia and Mexico. Tob Control.

[32] Miller CL, Quester PG, Hill DJ, Hiller JE (2011). Smokers’ recall of Australian graphic cigarette packet warnings & awareness of associated health effects, 2005–2008. BMC Public Health.

[33] Hoek J, Gendall P, Eckert C, Rolls K, Louviere J (2016). A comparison of on-pack Quitline information formats. Tob Control.

[34] Baskerville NB, Hayward L, Brown KS, Hammond D, Kennedy RD, Campbell HS (2015). Impact of Canadian tobacco packaging policy on quitline reach and reach equity. Prev Med.

[35] Baskerville NB, Brown KS, Nguyen NC, Hayward L, Kennedy RD, Hammond D (2016). Impact of Canadian tobacco packaging policy on use of a toll-free quit-smoking line: an interrupted time-series analysis. CMAJ Open.

[36] Park J, Minh LN, Shin SH, Oh JK, Yun EH, Lee D (2019). Influence of new tobacco control policies and campaigns on Quitline call volume in Korea. Tob Induc Dis.

[37] Dubray J, Schwartz R, Chaiton M, Connor S, Cohen JE (2015). The effect of MPOWER on smoking prevalence. Tob Control.

[38] Feliu A, Filippidis FT, Joossens L, Fong GT, Vardavas CI, Baena A (2019). Impact of tobacco control policies on smoking prevalence and quit ratios in 27 European Union countries from 2006 to 2014. Tob Control.

[39] Han MA (2019). The price of tobacco and its effects on smoking behaviors in Korea: the 2015 Korea Community Health Survey. Prev Med.

[40] Wilkinson AL, Scollo MM, Wakefield MA, Spittal MJ, Chaloupka FJ, Durkin SJ (2019). Smoking prevalence following tobacco tax increases in Australia between 2001 and 2017: an interrupted time-series analysis. Lancet Public Health.

[41] Ahmad S, Franz GA (2008). Raising taxes to reduce smoking prevalence in the US: a simulation of the anticipated health and economic impacts. Public Health.

[42] Levy DT, Wen CP, Chen TY, Oblak M (2005). Increasing taxes to reduce smoking prevalence and smoking attributable mortality in Taiwan: results from a tobacco policy simulation model. Tob Control.

[43] Agaku IT, Filippidis FT, Vardavas CI (2015). Effectiveness of text versus pictorial health warning labels and predictors of support for plain packaging of tobacco products within the European Union. Eur Addict Res.

[44] Noar SM, Hall MG, Francis DB, Ribisl KM, Pepper JK, Brewer NT (2016). Pictorial cigarette pack warnings: a meta-analysis of experimental studies. Tob Control.

[45] Huang J, Chaloupka FJ, Fong GT (2014). Cigarette graphic warning labels and smoking prevalence in Canada: a critical examination and reformulation of the FDA regulatory impact analysis. Tob Control.

[46] Carter BD, Abnet CC, Feskanich D, Freedman ND, Hartge P, Lewis CE (2015). Smoking and mortality—beyond established causes. N Engl J Med.

[47] Lucas M, O’Reilly EJ, Mirzaei F, Okereke OI, Unger L, Miller M (2013). Cigarette smoking and completed suicide: results from 3 prospective cohorts of American adults. J Affect Disord.

[48] Poorolajal J, Darvishi N (2016). Smoking and suicide: a meta-analysis. PLoS One.

[49] Berlin I, Covey LS, Donohue MC, Agostiv V (2011). Duration of smoking abstinence and suicide-related outcomes. Nicotine Tob Res.

[50] Hughes JR (2008). Smoking and suicide: a brief overview. Drug Alcohol Depend.

[51] Lawn SJ, Pols RG, Barber JG (2002). Smoking and quitting: a qualitative study with community-living psychiatric clients. Soc Sci Med.

[52] Clancy N, Zwar N, Richmond R (2013). Depression, smoking and smoking cessation: a qualitative study. Fam Pract.

[53] Ziedonis D, Hitsman B, Beckham JC, Zvolensky M, Adler LE, Audrain-McGovern J (2008). Tobacco use and cessation in psychiatric disorders: National Institute of Mental Health report. Nicotine Tob Res.

[54] Brown RA, Kahler CW, Zvolensky MJ, Lejuez CW, Ramsey SE (2001). Anxiety sensitivity: relationship to negative affect smoking and smoking cessation in smokers with past major depressive disorder. Addict Behav.

[55] Gemma T, Ann M, Alan G, Amanda F, Nicola L-H, Paul A (2014). Change in mental health after smoking cessation: systematic review and meta-analysis. BMJ..

[56] John U, Meyer C, Rumpf H-J, Hapke U (2004). Smoking, nicotine dependence and psychiatric comorbidity—a population-based study including smoking cessation after three years. Drug Alcohol Depend.

[57] Davey Smith G, Hemani G, Jones HJ, Lawn RB, Munafò MR, Richmond RC (2020). Evidence for causal effects of lifetime smoking on risk for depression and schizophrenia: a Mendelian randomisation study. Psychol Med.

[58] Stepankova L, Kralikova E, Zvolska K, Pankova A, Ovesna P, Blaha M (2017). Depression and smoking cessation: evidence from a smoking cessation clinic with 1-year follow-up. Ann Behav Med.

[59] Goodwin RD, Eaton WW (2005). Asthma, suicidal ideation, and suicide attempts: findings from the baltimore epidemiologic catchment area follow-up. Am J Public Health.

[60] Rønnstad ATM, Halling-Overgaard A-S, Hamann CR, Skov L, Egeberg A, Thyssen JP (2018). Association of atopic dermatitis with depression, anxiety, and suicidal ideation in children and adults: a systematic review and meta-analysis. J Am Acad Dermatol.

[61] Postolache TT, Komarow H, Tonelli LH (2008). Allergy: a risk factor for suicide?. Curr Treat Options Neurol.

[62] van der Vaart H, Postma DS, Timens W, Ten Hacken NHT (2004). Acute effects of cigarette smoke on inflammation and oxidative stress: a review. Thorax.

[63] Chang HB, Munroe S, Gray K, Porta G, Douaihy A, Marsland A (2019). The role of substance use, smoking, and inflammation in risk for suicidal behavior. J Affect Disord.

[64] Odebrecht Vargas H, Vargas Nunes SO, Pizzo de Castro M, Cristina Bortolasci C, Sabbatini Barbosa D, Kaminami Morimoto H (2013). Oxidative stress and lowered total antioxidant status are associated with a history of suicide attempts. J Affect Disord..

[65] Oh Y (2021). The National Health Plan 2030: its purpose and directions of development. J Prev Med Public Health.

